# In-vitro determination of antimicrobial activities of *Eruca sativa* seed oil against antibiotic-resistant gram-negative clinical isolates from neonates: a future prospect

**DOI:** 10.1186/s12906-022-03710-1

**Published:** 2022-08-27

**Authors:** Rasha H. Bassyouni, Zeinat Kamel, Alkassem Ahmed Algameel, Ghada Ismail, Sylvana N. Gaber

**Affiliations:** 1 Department of Medical Microbiology and Immunology, Faculty of Medicine, Fayoum University, Fayoum, Egypt; 2 Department of Botany, Faculty of Science, Cairo University, Giza, Egypt; 3 Department of pediatrics, Faculty of Medicine, Fayoum University, Fayoum, Egypt; 4 Department of Clinical Pathology, Faculty of Medicine, Ain Shams University, Cairo, Egypt

**Keywords:** Neonates, Asymptomatic bacteriuria, Arugula oil, Antiseptic

## Abstract

**Background:**

The aim of this study is investigate the antimicrobial effect of plant oils against bacterial strains isolated from neonatal asymptomatic bacteriuria (ABU) and to evaluate the antiseptic effect of the most potent one.

**Methods:**

The antimicrobial effect of 17 plant oils were tested against 15- gram-negative bacterial strains recovered from cases of neonatal ABU (11 *Escherichia. coli*, 3 *Klebsiella pneumonia*, and 1 *Pseudomonas aeruginosa)* using the agar well diffusion method. The micro-dilution method was performed to investigate the minimum inhibitory concentrations (MIC) and the minimum bactericidal concentrations (MBC) in concentrations ranging from 1.95 μg/ml to 500 μg/ml. The evaluation of the antiseptic activity of the *Eruca sativa* (arugula) seed oil was investigated using time-kill assay in concentrations ranging from 50 μg/ml to 0.195 μg/ml.

**Results:**

All tested oils showed variable antimicrobial activities against the tested strains. Arugula, wheat germ, cinnamon, parsley, dill, and onion oils were the most active oils. Among them, arugula oil was the most active oil with MIC_50_ and MIC_90_ were 3.9 μg/ml and 31.3 μg/ml respectively. MBC_50_ and MBC_90_ of arugula oil were 15.6 μg/ml and 125μg/ml respectively. The time-kill assay of arugula oil indicated that a concentration of 100 μg/ml completely killed nine of the tested strains after 10 min and reduced the CFU/ml of the rest of the strains by 3 log^10^ at the same time interval.

**Conclusion:**

Arugula seed oil could be a potentially used as an antiseptic especially for neonates.

## Introduction

Asymptomatic bacteriuria (ABU) is the finding of positive cultures of the same pathogen from two successive urine samples, without urinary symptoms. The prevalence of ABU is assessed to be 1% in older children, 3% in school-aged children, and 1% in full-term infants; this number increased up to 10.7% in febrile infants in less than 30 days [1, 2]. Infants are usually more likely to be infected than others especially with underlying renal abnormality, although most cases occur without any abnormalities [3]. The bacteria that inhabit the distal gastrointestinal tract and colonize the perianal area most likely attributed to urinary tract infections (UTI). Bacteria may enter the urinary tract when an infant has an unclean diaper or is wiped from back to front*. E. coli* usually causes a child’s first infection but, other gram-negative bacilli such as *klebsiella* spp. and *Enterococcus* spp. may also cause infection [4].

In the pediatric population, recent reports have shown that antibiotics not effective in treating ABU unless they have undergone invasive urologic procedures or received a renal transplant [1]. The use of prophylactic antibiotics to prevent recurrent UTI increases the risk for emergence of resistant organisms [5]. Therefore, it is critical to develop new compounds with novel mechanisms of action and determine their safety when used as a topical antiseptic to prevent bacteriuria in the pediatric population. Plants have been scientifically proven to have novel compounds with antimicrobial properties which are of great therapeutic significance [6]. Extracted essential oils (EOs) from plants, are interesting natural products [7]. EOs have been used in the early years for numerous purposes, until today in the medical field [8]. The use of some EOs as an alternative to antimicrobial agents or in combination with antibiotics has recently attracted considerable interest among researchers [7, 9]. Health and Human Services as well as Public Health Services had recognized essential oils as safe substances including compounds extracted from some EOs that can be used as antibacterial additives to food [10]. In systemic infections, plant oils have a weak action compared with the antibiotics and are poorly absorbed by intestine. However, despite these undesirable facts, numerous EOs have been used for treating localized bacterial infections [11]. *Cymbopogon citratus* oil has shown respectable antimicrobial activities against resistant microorganisms isolated from neonatal intensive care units both in-vitro and in-vivo and may serve as an alternative agent in the treatment of microorganisms that are resistant to many groups of antibiotics [12].

The aim of this study is to investigate the antimicrobial effect of 17 plant oils against bacterial strains isolated from asymptomatic neonatal bacteriuria and evaluate the antiseptic effect of the most potent one.

## Material and methods

The objectives and procedures of the present study were permitted by the Institutional Ethics Committee (Fayoum University Ethics committee) NO: R106. The study has been conducted in compliance with Helsinki’s medical guidelines. Written informed consent of all participants’ parents was obtained after they were briefed about the study’s objectives.

This study was performed on 240 neonates. Samples were supplied from the neonatal department to the Medical Microbiology and Immunology Department, Faculty of Medicine, Fayoum University, Egypt. The study was conducted from September 2020 to May 2021.

### Identification of isolated bacteria

The identification of isolated bacteria was performed according to standard microbiological methods [13]. Gram-negative bacilli were further identified by the Microbact™ (12A +12B combined) gram-negative identification system (Oxoid, Basingstoke, UK).

### Antimicrobial susceptibility tests

Antibiotic susceptibility testing was performed by the Kirby-Bauer disc diffusion method according to the Clinical Laboratory Standard Institute (CLSI) guidelines [14]. The antibiotic discs used were as follows: Third-generation cephalosporins; ceftriaxone (30 μg), cefpodoxime (30 μg). cefotaxime, (30 μg), ceftazidime (30 μg)), Fourth-generation cephalosporin; cefepime (30 μg), Monobactam; aztreonam (30 μg), β-lactamase inhibitor; Piperacillin-tazobactam, (100/10 μg), Quinolone; ciprofloxacin (5 mg), Aminoglycoside;amikacin (30 μg) and Carbapenem; meropenem (10 μg) (Oxoid Ltd., UK). For quality control, *E. coli* ATCC 25922 was used.

Screening of isolates for β-lactamase production was tested using the Kirby-Bauer disk diffusion method [14].

### Phenotypic confirmatory tests for extended Spectrum β lactamases (ESBL) production

Isolates with one or more of the following criteria were considered to be potential ESBLs and were listed for confirmation of ESBL production by the combined disks method according to the CLSI guidelines [14]. The criteria included an inhibition zone of ≤22 mm for ceftazidime, and or ≤ 27 mm for cefotaxime, and or ≤ 25 mm for ceftriaxone and/or ≤ 17 mm for cefpodoxime and or aztreonam ≤27 mm.

The combined disk method was performed as follows: cefotaxime (30 μg) disk with or without clavulanate (10 μg) were used. A lawn culture was made on the Mueller Hinton Agar plate (Oxoid Ltd., UK) and the disks were placed 25 mm apart (center to center) from each other and incubated aerobically overnight at 37 °C. A difference in the zone of inhibition of more than 5 mm of either of the cephalosporin disks and their clavulanate containing disks indicates the production of ESBL. *Klebsiella pneumoniae* ATCC 700603 were used as the control strains.

### Phenotypic screening for carbapenemases producing strains

Isolates resistant or intermediately resistant to meropenem (inhibition zone ≤23 mm) were subjected to modified carbapenem inactivation test [15].

### Screening of the antimicrobial activity of plant oils

The antimicrobial effect of 17 plant oils was tested against isolates; parsley (*Petroselinum crispum*) seed oil, flaxseed (*Linum usitatissimum* L.) oil, *nigella sativa* seed oil, sesame (*Sesamum indicum L*.) seed oil, dill (*Anethum graveolens* L.) herb oil, cinnamon Bark (*Cinnamomun zeylanicum*) oil, onion (*Allium cepa L*.) seed oil, arugula (*Eruca sativa*) seed oil, camphor (*Cinnamomum camphora*) crystal oil, sage)*Salvia officinalis*(oil, basil (*Ocimum basilicum L*.) leaf oil, thyme (*Thymus vulgaris L*.) seed oil, mint (*Mentha spicata L*) seed oil, wheat germ oil, coriander (*Coriandrum sativum L*.) seed oil, garlic (*Allium sativum L*.) head oil and caraway (*Carum carvi)* seed oil) (El Hawag Natural Oils Co, Nasr City, Cairo, Egypt) by agar well-diffusion method as previously described by *Valizadeh* et al. [16] with slight modification. Briefly*,* freshly prepared inoculum (10^7^ CFU/ ml) was streaked all over the surface of the Muller Hinton agar (Oxoid Ltd., UK), the wells were made in the medium with the help of a sterile cork-borer 6-mm diameter, 50 μl of each oil was added to each well, and 50 μl of sterile broth was added to one well as a negative control. The experiment was conducted in triplicate. The plates were allowed to stand for 1 h at room temperature for the diffusion of the essential oil into the agar then incubated at 37 °C for 24 h. The diameters of the zones of inhibition were measured and reported.

### Determination of minimum inhibitory concentration (MIC) and minimum bactericidal concentration (MBC)

The micro-dilution method was used to investigate the MIC and MBC of arugula, wheat germ, cinnamon, parsley, dill and onions oils which were the most active oils against the tested strains with concentrations ranging from 500 μg/ml to 1.95 μg/ml (two-fold dilutions) as described by *Semeniuc* et al. [17].

### Time kill activity of arugula seed oil and chlorohexidine gluconate

The arugula oil was further evaluated as it showed the lowest MIC_50_, MIC_90_, MBC_50_, and MBC_90_ against the tested strains. The evaluation of its time-kill activity as described by *Zu* et al. [18] was performed as an indicator of antiseptic activity in a concentration ranging from 50 μg /ml to 0.195 μg /ml (2 fold dilutions). Counts of viable colonies were carried out at different intervals (0, 5, and 10 minutes) after incubation for 24 h at 37 °C. The kill curves were plotted with time against the logarithm of the viable colony counts (CFU/ml). A bactericidal effect is considered when a 3 log_10_ decrease in the CFU/ml or a 99.9% kill over a specified time is observed [19]. Chlorohexidine gluconate (2%) one of the widely used antiseptics in neonatology units (Sigma- Aldrich, St. Louis, MO) [4] was also tested against the isolated strains by time kill curve.

### Gas chromatography–mass spectrometry analysis of arugula seed oil

Gas chromatography–mass spectrometry analysis (GC-MS) was conducted using the Agilent auto system 7890B GC-MS equipped with the HB-5MS capillary column (5% phenyl–95% dimethyl polysiloxane, 30 m × 0.25 mm × 0.25 μm). The carrier gas was helium with a flowrate of 1 ml/min. The oven temperature was maintained at 60 °C for 5 min, programmed to 240 °C at a rate of 3 °C/min, and then fixed at 240 °C for 10 min. The injector, ion source, GC–MS interface and mass detector temperature were maintained at 230 °C, 200 °C, 270 °C, and 150 °C, respectively. Mass spectra were taken at 70 eV. The scan duration was 0.25 sec, and the mass range was 50–500 Da.

1 μl of the sample was injected at a split ratio of 1:50. The ionization of the sample was done in the EI ion source at 70 eV and the acquisition mass range was set at 35–500 *amu*. The identification of components was based on a comparison of their mass spectra (using molecular ion (M+) peak and the m/z values) with those provided in the mass spectra library NIST (2011). The relative peak area percentages were used to report the abundance of a compound in the oil [20].

#### Statistical analysis

Statistical analysis was performed using (Statistical Package for the Social Science) version 16 for Windows. Data were statistically described in terms of frequencies and percentages. For quantitative data, ranges were calculated. Qualitative data were presented as numbers and percentages.

## Results

Our results revealed, 17 isolates were isolated from 240 neonates: two of them gram positive isolates (*staph aureu*s) were sensitive to all tested antibiotics (not included in the study), and 15 g negative isolates: 11/15 isolates were *E. coli*, 3/15 isolates were *Klebsiella pneumonia (K. pneumonia),* and 1 isolate was *Pseudomonas aeruginosa (P. aeruginosa).* Table 1 shows the antibiotic susceptibility patterns of the resistant isolates.

**Table 1:** The antibiotic susceptibility patterns of the tested bacteria

**Isolate code**	**Inhibition zone in mm (susceptibility pattern)**									
	**Meropenem**	**Ceftriaxone**	**Cefpodoxime**	**Cefotaxime<**	**Aztronam<**	**Amikacin**	**Piperacillin- tazobactam**	**Ceftazidime<**	**Ciprofloxacin**	**Cefepime**
**E1**	**24 (S)**	**0 (R)**	**0 (R)**	**0 (R)**	**22 (S)**	**17(S)**	**22(S)**	**17(R)**	**22(S)**	**25(S)**
**E2**	**23 (S)**	**0(R)**	**0 (R)**	**0 (R)**	**27 (s)**	**13(R)**	**23(S)**	**15(R)**	**25(S)**	**28(S)**
**E3**	**24 (S)**	**0(R)**	**0 (R)**	**0 (R)**	**22(S)**	**18(S)**	**26(S)**	**23(S)**	**30(S)**	**28(S)**
**E4**	**30(S)**	**22(I)**	**21 (S)**	**22(R)**	**28(S)**	**23(S)**	**28(S)**	**24(S)**	**30(S)**	**30(S)**
**E5**	**28(S)**	**26(S)**	**23 (S)**	**21(R)**	**24(S)**	**24(S)**	**30(S)**	**24(S)**	**32(S)**	**27(S)**
**E6**	**28 (S)**	**25(S)**	**22 (S)**	**29(S)**	**24(S)**	**22(S)**	**25(S)**	**21(S)**	**30(S)**	**29(S)**
**E7**	**29(S)**	**26(S)**	**23(S)**	**22(R)**	**27(S)**	**25(S)**	**26(S)**	**24(S)**	**30(S)**	**27(S)**
**E8**	**24 (S)**	**11(R)**	**0 (R)**	**15 (R)**	**22(S)**	**18(S)**	**24(S)**	**18(I)**	**25(S)**	**25(S)**
**E9**	**26 (S)**	**22(I)**	**15(R)**	**0(R)**	**24(S)**	**16(I)**	**26(S)**	**27(S)**	**24(S)**	**26(S)**
**E10**	**23 (S)**	**0(R)**	**0 (R)**	**0(R)**	**20(I)**	**18(S)**	**22(S)**	**17(R)**	**22(S)**	**25(S)**
**E11**	**27(S)**	**23(S)**	**23 (S)**	**22(R)**	**28(S)**	**27(S)**	**28(S)**	**26(S)**	**29(S)**	**30(S)**
**K1**	**23(S)**	**13(R)**	**9(R)**	**19(R)**	**21(S)**	**17(S)**	**23(S)**	**22(S)**	**22(S)**	**27(S)**
**K2**	**20(I)**	**10(R)**	**0(R)**	**10(R)**	**18(I)**	**14(R)**	**24(S)**	**17(R)**	**26(S)**	**16(R)**
**K3**	**21(I)**	**0(R)**	**0 (R)**	**0(R)**	**20(I)**	**17(S)**	**22(S)**	**21(S)**	**26(S)**	**17(R)**
**P**	**0(R)**	**0(R)**	**0 (R)**	**0(R)**	**15(R)**	**17(S)**	**13(R)**	**12(R)**	**18(I)**	**14 (R)**

***E ****Escherichia coli*, ***K ****Klebsiella pneumonia,****P ****Pseudomonas aeruginosa*, ***S*** Susceptible, ***I*** Intermediate resistant, ***R*** Resistant

Although 14 isolates were ESBL producers by the disk diffusion method, the combined disk diffusion test revealed that only 10 strains were ESBL producers; 6 *E. coli*, 3 *K. pneumonia*, and 1 *P. aeruginosa strains.* Moreover, three isolates were resistant or intermediately resistant to meropenem by the disk diffusion method and the modified carbapenem inactivation test revealed that those three isolates were carbapenemase-producing isolates; *2 K. pneumonia* and 1 *P. aeruginosa.*

Table 2 shows the evaluation of the antimicrobial activity of 17 essential oils against bacterial isolates are shown in Table 2. All tested oils showed variable antimicrobial activities against tested strains. Arugula, wheat germ, cinnamon, parsley, dill and onion oils were the most active oils. Arugula oil induces more activities, while sesame and camphor oils induce less activities against the tested strains.

**Table 2:** The frequency of the tested strains inhibited by plant oils using the well diffusion method

**Tested oils**	**Number of inhibited strains according to zone of inhibition (total no. =15)**			
	**0–10 mm**	**11–20 mm**	**21–30 mm**	**31–40 mm**
**Parsley**	1	11	3	0
**Flaxseed**	8	7	0	0
***Nigella sativa***	11	4	0	0
**Sesame**	15	0	0	0
**Dill**	2	12	1	0
**Cinnamon**	2	10	3	0
**Arugula**	0	0	**3**	**12**
**Onion**	3	7	5	0
**Camphor**	15	0	0	0
**Sage**	10	5	0	0
**Basil**	7	8	0	0
**Thyme**	6	9	0	0
**Mint**	4	11	0	0
**Wheat germ**	2	9	4	0
**Caraway**	11	4	0	0
**Coriander**	14	1	0	0
**Garlic**	13	2	0	0

Table 3 shows the evaluation of the MIC, and MBC of arugula, wheat germ, cinnamon, parsley, dill, and onion oils which are the most active oils against the tested strains. All tested oils were active against the tested strains in very low concentrations. Arugula oil was the most active oil with MIC_50_ and MIC_90_ were 3.9 μg/ml and 31.3 μg/ml respectively. MBC_50_ and MBC_90_ of arugula oil were 15.6 μg /ml and 125 μg /ml respectively (Table 4).

**Table 3:** The minimum inhibitory concentrations (MIC) and minimum bactericidal concentrations (MBC) of six essential oils against the tested bacteria (μg/ml)

**Isolate code**	**Arugula oil**		**Wheat germ oil**		**Cinnamon oil**		**Parsley oil**		**Onion oil**		**Dill oil**	
	**MIC**	**MBC**	**MIC**	**MBC**	**MIC**	**MBC**	**MIC**	**MBC**	**MIC**	**MBC**	**MIC**	**MBC**
**E1**	7.8	31.25	31.25	62.5	31.25	125	15.6	62.5	62.5	125	31.25	125
**E2**	3.9	15.6	31.25	62.5	62.5	251	15.6	62.5	62.5	250	31.25	125
**E3**	3.9	15.6	31.25	62.5	62. 5	250	62.5	125	62.5	125	31.25	125
**E4**	1.95	7.8	7.8	31.25	31. 25	125	7.8	31.25	15.6	31.25	15.6	62.5
**E5**	1.95	7.8	15.6	31.25	31. 25	125	7.8	31.25	15.6	31.25	15.6	62.5
**E6**	1.95	7.8	15.6	31.25	31. 25	125	7.8	31.25	15.6	31.25	15.6	31.25
**E7**	1.95	7.8	7.8	31.25	31. 25	125	15.6	31.25	15.6	31.25	31.25	62.5
**E8**	3.9	15.6	31.25	62.5	31.25	250	15.6	62.5	62.5	250	31.25	125
**E9**	3.9	15.6	31.25	62.5	62.5	250	31.25	125	62.5	125	31.25	125
**E10**	3.9	15.6	31.25	62.5	31.25	125	15.6	62.5	62.5	125	31.25	250
**E11**	1.95	7.8	15.6	31.25	31.25	125	7.8	31.25	15.6	62.5	15.6	31.25
**K1**	31.25	.125	7.8	31.25	250	500	15.6	62.5	31.25	62.5	31.25	125
**K2**	31.25	125	15.6	125	250	500	15.6	125	62.5	125	31.25	125
**K3**	31.25	125	15.6	62.5	250	500	15.6	62.5	31.25	62.5	31.25	125
**P**	31.25	125	7.8	125	15.6	62.5	62.5	125	31.25	62.5	31.25	125

***E ****E.coli*, ***K ****Klebsiella pneumonia, ****P ****Pseudomonas aeruginosa*

**Table 4:** The minimum inhibitory concentrations and minimum bactericidal concentrations of most active oils that inhibit/ kill 50 and 90% of tested bacteria (μg/ml)

**Tested oils**	**MIC**_50_	**MIC**_90_	**MBC**_50_	**MBC**_90_
**Arugula**	**3.9**	**31.25**	**15.6**	**125**
**Wheat germ**	15.6	31.25	62.5	125
**Cinnamon**	31.25	250	125	500
**Parsley**	15.6	62.5	62.5	125
**Onion**	31.25	62.5	62.5	250
**Dill**	31. 25	31.25	125	125

MIC 50: minimum inhibitory concentration that inhibits 50% of the tested isolates

MIC 90: minimum inhibitory concentration that inhibits 90% of the tested isolates

MBC 50: minimum bactericidal concentration that kills 50% of the tested isolates

MBC 90: minimum bactericidal concentration that kills 90% of the tested isolates

The time-kill assay of arugula oil against the tested strain revealed that a concentration of 100 μg /ml completely killed nine of the tested strains after 10 min and a 3 log_10_ decrease in the CFU/ml of the rest of the tested strains at the same time interval. A concentration of 50 μg /ml decrease the CFU/ml of most of the tested strains 3 log_10_ after 10 min. Other tested concentrations cannot exert the same effect, whereas chlorohexidine completely kills all tested strains after 5 min (Table 5).

**Table 5:** Time kill assay of different concentrations of arugula oil against the tested strains

**Isolate Code**	**Arugula oil (ug/ml)**						**Chlorhexidine 2%**
	**Time**	**100**	**50**	**25**	**12.5**	**6.25**	
**E1**	**0 min**	**5.69**	**5.74**	**5.74**	**5.76**	**5.77**	**5.69**
	**5 min**	**2.69**	**3.39**	**3.69**	**3.87**	**4.69**	**∞**
	**10 min**	**∞**	**2.69**	**3.30**	**3.69**	**3.69**	**∞**
**E2**	**0 min**	**5.71**	**5.75**	**5.76**	**5.76**	**5.77**	**5.77**
	**5 min**	**2.74**	**3.87**	**4.02**	**4.09**	**4.69**	**∞**
	**10 min**	**∞**	**2.97**	**3.30**	**3.39**	**3.60**	**∞**
**E3**	**0 min**	**5.69**	**5.74**	**5.77**	**5.77**	**5.84**	**5.69**
	**5 min**	**3.17**	**3.69**	**3.87**	**3.39**	**4.74**	**∞**
	**10 min**	**2.77**	**2.95**	**3.69**	**3.87**	**4.17**	**∞**
**E4**	**0 min**	**5.65**	**5.69**	**5.74**	**5.77**	**5.77**	**5.60**
	**5 min**	**2.99**	**3.69**	**3.98**	**4.39**	**4.47**	**∞**
	**10 min**	**∞**	**2.90**	**3.39**	**3.39**	**3.47**	**∞**
**E5**	**0 min**	**5.65**	**5.69**	**5.74**	**5.74**	**5.77**	**5.65**
	**5 min**	**3.30**	**3.54**	**3.09**	**4**	**4.07**	**∞**
	**10 min**	**∞**	**2.69**	**3.24**	**3.69**	**3.60**	**∞**
**E6**	**0 min**	**5.65**	**5.69**	**5.74**	**5.74**	**5.77**	**5.69**
	**5 min**	**2.99**	**3.69**	**3.98**	**3.39**	**3.47**	**∞**
	**10 min**	**∞**	**2.90**	**3.39**	**3.39**	**3.47**	**∞**
**E7**	**0 min**	**5.65**	**5.69**	**5.74**	**5.77**	**5.77**	**5.60**
	**5 min**	**2.99**	**3.69**	**3.98**	**4.39**	**4.47**	**∞**
	**10 min**	**∞**	**2.90**	**3.39**	**3.39**	**3.47**	**∞**
**E8**	**0 min**	**5.69**	**5.74**	**5.74**	**5.76**	**5.77**	**5.69**
	**5 min**	**2.68**	**3.39**	**3.69**	**3.87**	**4.69**	**∞**
	**10 min**	**∞**	**2.69**	**3.30**	**3.69**	**3.69**	**∞**
**E9**	**0 min**	**5.69**	**5.74**	**5.77**	**5.77**	**5.84**	**5.69**
	**5 min**	**3.17**	**3.69**	**3.87**	**3.39**	**4.74**	**∞**
	**10 min**	**2.77**	**2.95**	**3.69**	**3.87**	**4.17**	**∞**
**E10**	**0 min**	**5.71**	**5.75**	**5.76**	**5.76**	**5.77**	**5.77**
	**5 min**	**2.74**	**3.87**	**4.02**	**4.09**	**4.69**	**∞**
	**10 min**	**∞**	**2.97**	**3.30**	**3.39**	**3.60**	**∞**
**E11**	**0 min**	**5.65**	**5.69**	**5.74**	**5.74**	**5.77**	**5.65**
	**5 min**	**3.30**	**3.54**	**3.09**	**4**	**4.07**	**∞**
	**10 min**	**∞**	**2.69**	**3.24**	**3.69**	**3.60**	**∞**
**K1**	**0 min**	**5.69**	**5.74**	**5.77**	**5.77**	**5.84**	**5.69**
	**5 min**	**3.17**	**3.69**	**3.87**	**3.39**	**4.74**	**∞**
	**10 min**	**2.77**	**2.95**	**3.69**	**3.87**	**4.17**	**∞**
**K2**	**0 min**	**5.69**	**5.74**	**5.74**	**5.76**	**5.77**	**5.69**
	**5 min**	**2.69**	**3.39**	**3.69**	**3.87**	**4.69**	**∞**
	**10 min**	**2.39**	**2.69**	**3.30**	**3.69**	**3.69**	**∞**
**K3**	**0 min**	**5.71**	**5.75**	**5.76**	**5.76**	**5.77**	**5.77**
	**5 min**	**2.74**	**3.87**	**4.02**	**4.09**	**4.69**	**∞**
	**10 min**	**2.60**	**2.97**	**3.30**	**3.39**	**3.60**	**∞**
**P**	**0 min**	**5.69**	**5.75**	**5.76**	**5.74**	**5.77**	**5.69**
	**5 min**	**4.30**	**4.1**	**4.17**	**4.69**	**4.77**	**∞**
	**10 min**	**3.25**	**3.30**	**3.65**	**3.92**	**4**	**∞**

***E ****Escherichia coli*, ***K ****Klebsiella pneumonia, ****P ****Pseudomonas aeruginosa*, **∞** log 10 of 0

**Fig. 1:**
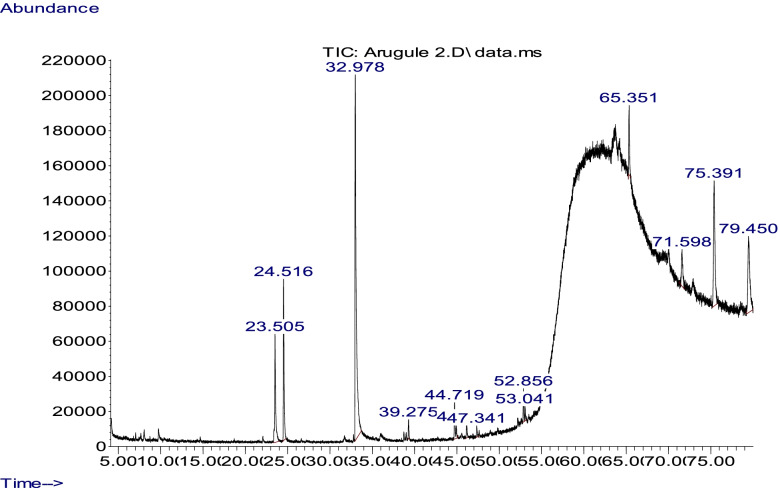
GC-MS profile of the *Eruca sativa* seed oil

The chromatogram (GC–MS) analysis of the arugula oil detects 12 compounds at various retention times (Table 6, Fig. 1). Sulforaphone nitrile, 5-methylthiopentanonitrile and 2-pentanonitrile represent 37, 93, 9, 67 and 8.43% respectively.

**Table 6:** Percentage of the compounds of Arugula seed oil as detected by GC-MS

**No.**	**RT**	**Compounds**	**Percentage of Total**
**1**	23.5	2-Pentanonitrile	8.43
**2**	24.5	5-Methylthiopentanonitrile	9.67
**3**	32.97	Sulforaphane nitrile	37.93
**4**	39.27	Phenindione	0.92
**5**	44.7	Eugenol	2.82
**6**	46.1	Docosane	0.96
**7**	52.8	Pentacosane	1.63
**8**	53.04	Hexadecadienal	0.76
**9**	65.3	Isophytole	4.2
**10**	71.59	Nonacosane	4.03
**11**	75.39	Squalene	15.35
**12**	79.45	Ergosterol	12.59

## Discussion

Newborns hospitalized in intensive care units are more liable to be colonized with hospital microorganisms, including antibiotic-resistant bacteria [12]. Effective skin disinfection with antiseptic agents is considered an important intervention to prevent or reduce healthcare-associated infections. A wide range of antiseptic preparations in varying combinations and concentrations has been used in neonatal units worldwide. However, good evidence is lacking, and the most appropriate and safe antiseptic to be used is still controversial [4]. Therefore, novel antiseptics with proven effectiveness and safety are necessary.

Plant extracted oils have long been used in alternative medicine and pharmaceutical therapies as well as in the preservation of food. Many of these oils have variable degrees of antimicrobial activities against different types of bacteria [12, 17, 21–23]. Accordingly, the present study investigated the antimicrobial effect of 17 plant extracted oils against 15 clinical bacterial strains isolated from patients with asymptomatic neonatal bacteriuria using the well diffusion method (11 *E. coli*, 3 *K. pneumonia*, and 1 *P. aeruginosa)*.

Our results revealed that all the tested oils had a considerable antimicrobial effect against all tested strains. The most active oils against the tested bacteria were arugula, cinnamon, wheat germ, dill, parsley, and onion oils. The findings of the present study are promising as most of the tested bacteria showed variable degrees of resistance against antibiotics; ESBL production was detected in six *E. coli*, three *K. pneumonia* (two of them were also carbapenemase-producers) and one *P. aeruginosa* strain showed both ESBL and carbapenemase production. In partial agreement with our results, *Bassyouni* et al. [24] screened the antimicrobial effect of 16 plant EOs against 36 g-positive and gram-negative bacterial strains isolated from the conjunctiva of patients submitted to cataract surgery, in which 15 of them showed antimicrobial activity against one or more bacterial strains. Dill oil, peppermint oil, and cinnamon oil were the most potent oils that showed a promising inhibitory activity against most the tested bacterial strains, but parsley oil was one of the least active oil against tested strains.

Likewise, in India *Khoobchandani* et al. [25] had examined the antimicrobial potential of numerous solvent extracts of arugula (*Eruca sativa*) (aerial and root) and seed oil against-antibiotic resistant gram-negative (*E.coli* ATCC 14169*, P. aeruginosa* MTCC 424 and *Shigella flexneri* MTCC 1457) and gram-positive (*Staphylococcus aureus* ATCC 6538 and *Bacillus subtilis MTCC* 441) bacteria. Among the several preparations, seeds oil was the most active, showing a maximum zone inhibition of 97% for gram-positive bacteria and 74 to 97% for gram-negative bacteria. Moreover, in Italy *Cannas* et al. [8] focused on the antimicrobial activity and in vitro cytotoxicity of 20 EOs to normal human conjunctival cells and reported that the tested oils showed no cytotoxic effect at very low concentrations. Rosmarinu sofficinalis, thymus vulgaris L. red thyme geraniolsel oils, and melaleuca alternifolia had good antimicrobial activity against gram-negative and gram-positive strains.

*Donadu* et al. [9] in Italy *have* also investigated the antimicrobial activity of two lavender EOs (lavanda grosso and lavanda sumian) against 16 multidrug-resistant *P.aeruginosa* strains isolated from clinical ocular samples and found that The EO derived from l.sumian had a lower antimicrobial activity when compared with L.grosso which was effective on 11 *P. aeruginosa* strains at a concentration of 8%.

Furthermore*, Ye* et al. [26] in China studied the antimicrobial effect of onion essential oil which has a potent inhibitory effect against standard bacterial strains (*S.aureus, E.coli*, *and B.subtilis*) as well as yeasts (*Rhodotorula glutinis*, *Saccharomyces cerevisiae*, and *Candida tropicalis*) and molds (*Aspergillus terreus, Aspergillus niger*, *and Monascus purpureus*) with inhibition zones ranging from 4.1 to 19.3 mm. Similarly, *Karimi* et al. [27] in Iran studied the antimicrobial activities of essential oil extracted from leaves and seeds of parsley against five pathogenic bacterial strains (*E.coli* ATCC 8739, *S.aureus* ATCC 25913*, Salmonella enterica* PTCC 1709*, Yersinia enterocolitica* PTCC 1477*,* and *Vibrio cholera* PTCC 1611). They noticed the inhibition zone diameters of the essential oil from leaves ranging from 12 to 14.5 mm and from seeds ranging from 9 to 11 mm. It was observed that different degrees of activities were detected in different studies. As the antimicrobial effectiveness of medicinal plants varies dramatically according to the phytochemical properties of plant families and subfamilies, it is common to detect a variation in antimicrobial efficacy even when using oils extracted from the same plant, but from two different geographical regions [28].

The present study investigated the MIC and MBC of the most potent oils against the tested strains. The results of MIC, MBC of arugula, wheat germ, cinnamon, parsley, dill and onion oils revealed that all tested oils were active against tested strains (all were gram-negative bacteria) in very low concentrations. Arugula oil was the most active oil with MIC_50_ and MIC_90_ were 3.9 μg /ml and 31.3 μg /ml respectively and MBC_50_ and MBC_90_ were 15.6 μg/ml and 125μg/ml respectively.

Gram-negative bacteria are more vulnerable to plant EOs than gram-positive bacteria [23, 29]. This can be clarified by that the outer membrane of gram-negative bacteria which is rich in lipopolysaccharide and more complex, can be limiting the diffusion of the hydrophobic compounds, whereas, in gram-positive bacteria no outer membrane which is bounded by a peptidoglycan wall not thick enough to resist small antimicrobial molecules, permitting them access to the cell membrane. Moreover, gram-positive bacteria might aid the hydrophobic molecules of plant oils to infiltrate owing to the existence of the lipophilic ends of lipoteichoic acid in the cell membrane [10, 30]. The present data is considered promising as it highlights the antimicrobial action of arugula oil which is usually consumed with food; against clinically isolated resistant gram-negative strains from neonatal ABU. Based on these results we tested the efficacy of arugula oil as an antiseptic. The most acceptable and appropriate method for detecting the bactericidal effect as well as a valid tool for investigating the dynamic interaction between the antimicrobial agent and the bacterial strain is the time-kill test. Moreover, a concentration-dependent antimicrobial agent and/or a time-dependent factor is revealed by the time-kill test [10]. The present study has found that chlorohexidine (2%) succeeded to kill all the tested strains after 5 min, while arugula oil with a concentration of 100 μg /ml resulted in promising activates as it completely killed nine of the tested strains after 10 min and a 3 log_10_ decrease in the CFU/ml of the rest of the tested strains at the same time interval. It was observed that 100 μg/ml arugula oil needs more time than chlorohexidine to exert the same effect. However, when considering the safety of arugula oil versus the side effects reported for chlorohexidine; such as skin irritation, photosensitization, hypersensitivity, and anaphylaxis and the fact that preterm infants have immature skin with increased permeability, neurological symptoms and metabolic limitations could be possible which may result in a decrease in drug clearance [31]. Arugula oil could be safer than chlorohexidine as a neonatal antiseptic.

Although previous studies had reported the antimicrobial activities of arugula oil; most of them were conducted on standard strains [32, 33]. The present study is the first to investigate the effectiveness of arugula oil as an antiseptic especially against organisms isolated from such a vulnerable group of patients. The high activity of arugula oil as an antimicrobial agent in the present study could be explained by the high content of antimicrobial compounds detected by GC-MS analysis; sulforaphone nitrile, 5-methylthiopentanonitrile, and 2-pentanonitrile which represented 37, 93%, 9, 67, and 8.43% of the oils respectively. Previous studies have shown that pure sulforaphane has a broad antimicrobial spectrum effect against both gram-positive and gram-negative bacteria [34–38]. *Hichri* et al. [39] attributed the high antimicrobial activity of *E. longirostris* of roots, fruits and stems to their isothiocyanate and nitrogen components such as erucin and 5-(methylthio)-pentanenitrile. Furthermore, they found synergistic antimicrobial effects of the sulphur and the nitrogen compounds present in the same plant.

Arugula oil is used as a hair tonic to prevent hair loss, in the treatment of burns, and as an ointment for the treatment of eye infection, as well as digestive problems [32]. Moreover, it also has anticancer and antioxidant effects [40–42]. Many studies have investigated the application of arugula oil on hair and skin for its benefits; *Shatalebi* et al. [43] studied the application of oil/water hair wax formulas for hair growth composed of the ethanolic extract of *E. sativa* seed oil, with the addition of thickening agents in variable concentrations. The tested formulations were evaluated and compared with branded brand marketed products. Best results were observed with the formula containing 10% *E. sativa* seed oil and 10% ethanolic extract of propolis. This formula increased the hair length and weight of the newly grown hair, and improved the percentage phase of hair follicles. *Sanad* et al. [44] formulated and evaluated the enriched garlic and *E. sativa* oil stable cream with antibacterial activity. The preparation of different formulations was performed using different concentrations of two surface-active agents. Cream prepared with 2% surfactant mixture showed a well-designed formulation and the best antimicrobial activity with no skin irritation. *Taha* et al [45] formulated a new herbal antifungal hair cream containing *E. sativa* and garlic oils, active on pathogenic fungi (Trichophyton mentagiophytes, Malassezia furfur,and Microsporum canis Bodin) using different ratios of a non-ionic surfactant. The highest permeation rate of alliin in the presence of *E. sativa* oil which is necessary for antifungal activity was obtained with a formula of 4% concentration of Span and Brij. *Eruca sativa* seed oil is used for cooking and is reported to exhibit an antibacterial and antifungal activities [32, 33]*.* It is also used as a lubricant [46]*.* For all these benefits it can be used safely for the prevention of ABU as an antiseptic, as well as for the prevention and treatment of diaper dermatitis.

Diaper dermatitis is one of the most prevalent skin conditions that infants usually suffer from and caregivers should manage theme in the first year of their life. Nonmedical skincare practices and products that support healthy skin barrier function can prevent diaper dermatitis or alleviate the condition in most cases. The usage of barrier lubricants and improved diaper technology usually results in keeping moisture and irritants away from an infant’s delicate skin [47]*.* In conclusion, arugula oil shows a powerful antimicrobial effect against multidrug-resistant gram-negative bacteria isolated from neonatal ABU. It could be a promising alternative to chlorohexidine gluconate as an antiseptic in neonatal intensive care units because it is a safe and natural product.

## Data Availability

All data generated or analyzed during this study are included in this published article.
